# ViTT: Vision Transformer Tracker

**DOI:** 10.3390/s21165608

**Published:** 2021-08-20

**Authors:** Xiaoning Zhu, Yannan Jia, Sun Jian, Lize Gu, Zhang Pu

**Affiliations:** 1 School of Electronic Information Engineering, Beihang University, Beijing 100191, China; zhuxn@buaa.edu.cn (X.Z.); pungry@buaa.edu.cn (Z.P.); 2 School of Cyberspace Security, Beijing University of Posts and Telecommunications, Beijing 100876, China; jyn_1023@bupt.edu.cn (Y.J.); glzisc@bupt.edu.cn (L.G.)

**Keywords:** MOT, transformer, attention, backbone

## Abstract

This paper presents a new model for multi-object tracking (MOT) with a transformer. MOT is a spatiotemporal correlation task among interest objects and one of the crucial technologies of multi-unmanned aerial vehicles (Multi-UAV). The transformer is a self-attentional codec architecture that has been successfully used in natural language processing and is emerging in computer vision. This study proposes the Vision Transformer Tracker (ViTT), which uses a transformer encoder as the backbone and takes images directly as input. Compared with convolution networks, it can model global context at every encoder layer from the beginning, which addresses the challenges of occlusion and complex scenarios. The model simultaneously outputs object locations and corresponding appearance embeddings in a shared network through multi-task learning. Our work demonstrates the superiority and effectiveness of transformer-based networks in complex computer vision tasks and paves the way for applying the pure transformer in MOT. We evaluated the proposed model on the MOT16 dataset, achieving 65.7% *MOTA*, and obtained a competitive result compared with other typical multi-object trackers.

## 1. Introduction

The rapid development of deep neural networks (DNN) [1] has increased interest in applying deep learning to computer vision [2,3,4]. Supporting Multi-UAV operations through deep learning is a challenging and vital research area [5,6,7]. The realization of the above task depends on many essential computer vision technologies, among which multi-object tracking is an important one. MOT is used to identify and track a series of targets in a video sequence, but it faces challenges due to occlusion, complex backgrounds, and crowded scenes.

The most dominant and effective MOT methods [8,9,10,11] are based on the tracking-by-detection (TBD) paradigm, which involves tracking according to detection results. With respect to complexity, MOT can be divided into separate detection and embedding (SDE) and joint detection and embedding (JDE) models. For the SDE model, detectors [2,12,13] first locate all the objects in a single image via bounding boxes. Next, re-identification (Re-ID) [14,15] models extract embeddings for each bounding box. Finally, association modules link each object to one of the existing tracks through similarity metrics and complex association strategies [9] or create a new track. This method is accurate and progresses with the development of object detection and Re-ID, but using two separate models to detect objects and extract embedding ignores the potential structure that can be shared between them. The JDE model was created to address this shortcoming. Some recent work [16,17] combines object detection and Re-ID into a single network by multi-task learning, which greatly reduces MOT calculation.

**Figure 1:**
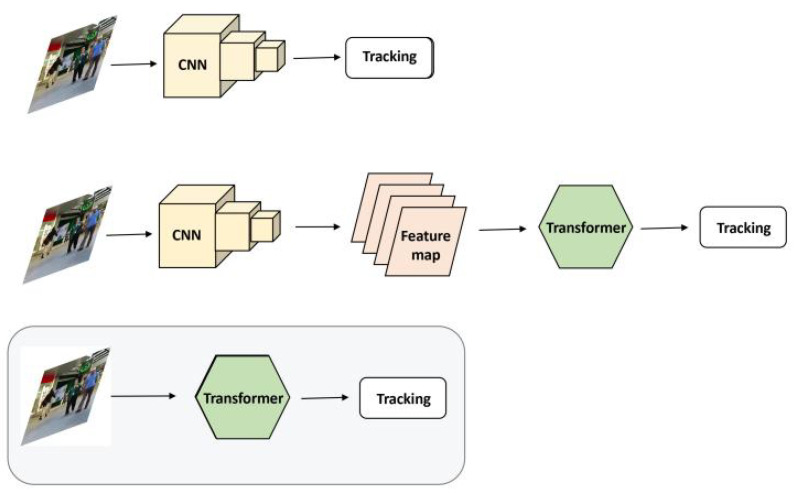
Comparison of different model architectures. Top diagram: In the traditional MOT model, a CNN is the foundation and is essential. Middle diagram: Although some works introduce a transformer into the computer vision models, they still need a CNN to extract the feature map first. Bottom diagram: Our work aims to explore the MOT model based on the transformer backbone. The identifiable image in this figure is from the public dataset MOT16.

Convolutional neural networks (CNNs) have always occupied a dominant position in traditional computer vision tasks [2,3,4,12]. The creation of transformers broadened the field. The transformer based on the attention mechanism [18] was initially applied to natural language processing, which replaced recurrent neural networks (RNN) and other cyclic networks and became the most dominant backbone. Recently, some research [19,20,21] has introduced transformers [22] into the computer vision field and achieved competitive results. DETR [19] is the first framework that successfully integrates a transformer into the central building block of a detection pipeline. It regards detection as a set of prediction problems and uses a transformer to predict a set of bounding boxes. Specifically, DETR uses a convolutional neural network to extract local information from an image and then uses a transformer to infer from the whole image and generate predictions. With the Vision Transformer [21], an image classification model based on a transformer was proposed, which takes images directly as the transformer’s input and outputs classification predictions through a multi-layer perception head. Specifically, it divides the original image into several patches, flattens them into a sequence, and feeds the sequence directly into the transformer encoder. This demonstrates that it is not necessary for image classification to depend on a CNN. Most existing visual models based on transformers use the “CNN + transformer” framework; that is, models extract feature maps using a CNN and then shape those maps as input into a transformer. The multi-object trackers based on the transformer backbone still need to be explored. A comparison of MOT models based on different architectures is shown in Figure 1.

Inspired by the aforementioned research, we apply a transformer to MOT and propose a new framework called Vision Transformer Tracker (ViTT). ViTT uses a transformer encoder as a backbone network, and it combines object detection and embedding extraction in a single shared network. Specifically, ViTT directly divides the image into small patches of fixed size, flattens each patch, and reshapes them into a 1D patch sequence. Next, the patch sequence is directly input into a transformer encoder after going through a patch embedding layer and a position embedding layer. Finally, the tracking head tracks according to the encoding result. Compared with “CNN + transformer” models [23,24], ViTT is more straightforward and more effective. Due to the locality found in convolution operators, CNNs have limitations in global context modeling. These limitations can only be solved by gradually expanding the receptive field by deepening the convolution layer. ViTT, however, can take advantage of the long-range dependence between patch sequences from the beginning using a self-attention mechanism. The code is available at: https://github.com/jiayannan/VITT.

## 2. Related Work

As our work is a multi-object tracker based on the transformer and multi-task learning, we introduce transformers and their applications in computer vision, and then we review the TBD paradigm and JDE method.

### 2.1. Transformers

A transformer is a new architecture based on the attention mechanism [22], which subverts the previous idea that sequence modeling means the use of RNN. It improves the shortcomings of RNN, such as slow training, and abandons the use of CNNs in previous deep learning tasks. Additionally, it replaces RNN in many DNN models [25,26,27,28,29]. Transformers aggregate the information found in a whole input sequence by introducing self-attention layers to encoders and decoders. An attention mechanism [18] is a neural network layer that can aggregate information from all input and enable the transformer to achieve a fast parallel. The transformer’s global computing power and perfect memory make it more suitable for long sequence processes. Moreover, the transformer’s depth can be increased to fully explore the characteristics of a DNN and to improve the accuracy of networks.

### 2.2. Transformers in Computer Vision

DETR [19] is the first model to introduce a transformer into computer vision successfully. It is based on the “CNN + transformer” architecture wherein a CNN first extracts feature maps as the transformer encoder’s input and obtains the result through the transformer decoder. DETR is a complete end-to-end model and does not require non-maximum suppression (NMS) as post-processing. It has some obvious disadvantages, such as slow convergence and poor detection effects for small objects. These shortcomings are mainly due to the self-attention operator’s enormous computational complexity when the input is high resolution. The deformable DETR [20] attempts to solve the aforementioned problems by introducing a deformable attention module to pay attention to the samples found in the feature maps. Both DETR and deformable DETR rely on CNNs to extract features. Vision Transformer [21] is the first visual model based on a pure transformer, and discards CNNs completely while achieving good image classification results. Although it is effective for image classification, there are no studies that show that the method can be extended to more complex visual tasks such as multi-object tracking. The Generative Pretraining from Pixels (iGPT) [30] directly applies a model based on the Generative Pretraining 2.0 (GPT-2) [28] to image pixels, which shows that an architecture based on a pure transformer is possible for some visual tasks. However, the input image resolution is very limited when using these methods. Other works explore the relationship between a self-attention module and a convolution layer [31] and analyze the limitations of convolutional neural networks [32,33].

### 2.3. Tracking-by-Detection

Most dominant trackers [8,9,10,11] follow the TBD paradigm which uses object detection models to identify object bounding boxes, uses Re-ID models to extract corresponding embeddings, and applies a Kalman filter [34] and the Hungarian algorithm [35] to associate objects and existing traces to achieve multi-object tracking. In other words, tracking can be regarded as the association between object bounding boxes, and the basis of association generally includes motion features and appearance features. These methods achieve good performance on public datasets. However, there is an obvious disadvantage: the object detection sub-models and Re-ID sub-models are separated, which results in the two computationally intensive networks significantly increasing the computational cost and complexity of the entire model. Some works propose the JDE method [16,17] to solve this problem. Specifically, they combine appearance embedding with a single-shot detector so that the models can output both detection results and corresponding embeddings in a shared network. There are also some works to simplify the association process: Tracktor [36], based on the faster RCNN [37] framework, directly takes the tracking results of previous frames as the Region Of Interest (ROI), and then applies the bounding boxes regression to provide the tracking results; Centertrack [38], based on centernet [39], predicts the offsets of objects relative to the previous frame while detecting objects.

## 3. Method

In this section, we introduce our model ViTT, which uses a transformer instead of a traditional CNN as the backbone network for feature extraction. Traditional multi-object trackers and existing multi-object trackers that combine a CNN and a transformer need to go through the CNN’s backbone network to extract feature maps. Our model inputs images directly into the transformer’s encoder and reconstructs the encoder’s outputs into final spatial feature maps to replace the convolution backbone network’s function. A CNN focuses on local information after downsampling several times, while a transformer can focus on global context information from the beginning. This model is based on multi-task learning, which outputs object classifications, locations, and embeddings simultaneously through a tracking head based on the encoder’s output feature maps. Our work demonstrates that a transformer can replace a CNN as a backbone network to complete complex computer vision tasks, thus paving the way for complex vision models based on a pure transformer.

The following is an accurate description of MOT. Given a video, i.e., an image frame sequence ${\left\{I\right\}}_{i=1}^{n}$, where n is the number of frames in the video, for each image frame $I_{i}\in R^{c\times w\times h}$ (i=1,…,n), where w, h and c are the width, height of images, and the number of images channels, trackers should give all objects $B\in R^{4\times k}$, their corresponding classification $C\in Z^{k}$, and serial number $N\in Z^{k}$, where k is the number of objects in the image. Since multi-object tracking is essentially the association between multiple objects in adjacent frames, MOT needs to number all objects in each frame. The same serial number marks the same object or track. When a new object appears, trackers give it a new serial number. When an old object reappears, trackers mark it with the original serial number. However, a DNN cannot directly output serial numbers in a practical way. Most existing multiple-object trackers are based on the outputs of DNNs. Networks only need to output each object’s location and other attributes associated with each object, including motion information and appearance information. Our model’s multi-task learning outputs the corresponding appearance embeddings $E\in R^{d\times k}$ while outputting the detection results, where d is the dimension of embedding vectors. Finally, MOT can be achieved by an association strategy such as the Hungarian algorithm.

### 3.1. Architecture

**Figure 2:**
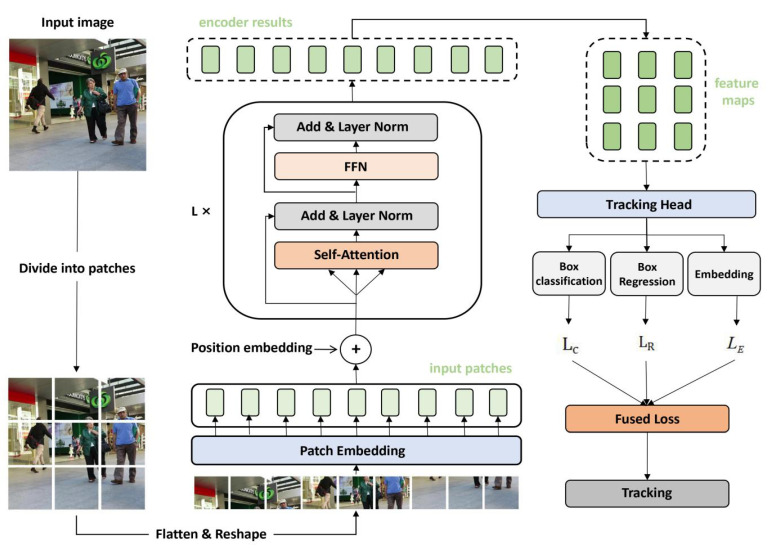
The overall architecture of ViTT. We use the transformer encoder to replace the traditional CNN to complete multi-object tracking directly based on the transformer encoder’s outputs. We reinterpret the encoder results into spatial feature maps and then output the object locations, classifications, and corresponding embeddings through a tracking head. The model inherits many advantages of the transformer and achieves strong performance. The identifiable image in this figure is from the public dataset MOT16.

An overview of the ViTT model is depicted in Figure 2, and it can be roughly divided into the following three phases. (1) Image serialization. Since the transformer’s standard input is a vector sequence, we need to shape input images to the desired format. Specifically, we divide each image into fixed-size patches, reshape these small patches into a patch sequence, and finally, convert the patch sequence into an embedding sequence via a patch embedding layer and a position embedding layer. (2) Transformer encoding. This part is the model’s backbone, which receives an embedding sequence as input and outputs an encoding sequence. We rearrange the output sequence back to the original position and view it as a group of feature maps after the downsampling directly. (3) Multi-task learning. The model produces locations, classifications, and appearance embeddings in a single shared network simultaneously through a tracking head based on the aforementioned feature maps. MOT is ultimately achieved through the association of objects from adjacent frames. A common association strategy is the Hungarian algorithm, which is not our focus. Next, we will introduce these parts in detail.

### 3.2. Image Serialization

Since a transformer needs a one-dimensional embedding vector sequence as input, we first need to obtain one-dimensional embedding vectors from three-dimensional input images. Specifically, we reshape each input image to a fixed size $I\in R^{w\times h\times 3}$, where w and h are the width and height of images, and the 3 is the number of image channels. We then divide images into patches $I_{p}\in R^{n\times (p^{2}\times 3)}$ of size p×p, where $n=\frac{wh}{p^{2}}$ is the number of patches. Finally, we flatten patches into embeddings ${\left\{p_{i}\right\}}_{i=1}^{n}$ of dimension d and add a learnable position embedding $E_{pos}$. The specific method is expressed as Equation (1):

(1) $$p_{i}=I_{p}^{i}E+E_{pos}.\;i=1,\ldots ,n,\;E\in R^{d\times (p^{2}\times 3)},\;E_{pos}\in R^{d\times n}$$

where E is a learnable linear projection.

Finally, we obtain the embedding vector sequence $z_{0}=[p_{1},\ldots ,p_{n}]$ as the input of the transformer encoder.

### 3.3. Encoder

The encoder of ViTT consists of L serial identical layers. Each layer consists of a multi-head self-attention (*MHA*) sublayer, a feed-forward network (*FFN*) sublayer, and several residual connections (we use “+” to indicate a residual connection calculation) and normalization layers (*LN*) following MHA and FFN. For all layers, the first layer takes an embedding sequence as input, and the others take the output of their upper layers as input, and the last layer outputs the final vector sequence $z_{L}$.

For each layer, the vector sequence first passes through an MHA sublayer, calculated as Equation (2):

(2) $$z_{l}^{\prime }=MHA(LN(z_{l-1}))+z_{l-1}.\;l=1,\ldots ,L$$

Following the MHA layer, the calculation process of the FFN sublayer is Equation (3):

(3) $$z_{l}=FFN(LN(z_{l}^{\prime }=))+z_{l}^{\prime }.\;l=1,\ldots ,L$$

where $z_{l-1}$ is the output of layer l−1, and $z_{l}$ is the output of layer l.

Each MHA sublayer contains H parallel self-attention heads, which allows the model to focus on different subspaces and expands its ability to focus on different locations. In this mechanism, the model keeps independent query Q, key K, and value V weight matrix for each head and obtains the output from multiple heads. These outputs are directly spliced together and then multiplied by a weight matrix W to aggregate them. The calculation process is expressed as Equation (4):

(4) $$MHA(Q,K,V)=Concat(h_{1},\ldots ,h_{H})W$$

where $h_{1},\ldots ,h_{H}$ are outputs of different heads. The calculation of self-attention in different heads is expressed as Equation (5):

(5) $$SelfAttention(Q,K,V)=soft\max(\frac{QK^{T}}{\sqrt{d_{k}}})V$$

where $d_{k}$ is the dimension of the key vector.

Each FFN sublayer consists of two linear layers and a dropout. The calculation process is Equation (6):

(6) $$FFN(z_{l}^{\prime })=FC_{2}(dropout(GELU(FC_{1}(z_{l}^{\prime })))).\;l=1,\ldots ,L$$

where GELU is an activation function.

### 3.4. Multi-Task Learning

Our model implements object detection and embedding extraction in a shared network. Specifically, bounding boxes, classifications, and appearance embeddings are generated simultaneously on the same feature map by the tracking head. Let the size of the input images be $O\times W_{in}\times H_{in}$, and the size of the output maps be $O\times W_{out}\times H_{out}$, where O is the dimension of output tensors. Then $\frac{W_{in}}{W_{out}}=\frac{H_{in}}{H_{out}}=R$, where R is the multiple of downsampling.

#### 3.4.1. Object Detection

The model’s object detection module is equivalent to a Region Proposal Network (RPN), whose function is to generate foreground candidate boxes and position offsets. Feature maps are first obtained through a backbone network, and each point on these feature maps is mapped to anchor boxes with different scale and aspect ratios in the original image. Then, feature maps are input into a RPN to classify whether anchor boxes belong to the foreground which results in the output of four predictive values representing the offset relative to the ground truth. It is important to note that we adjust the size and aspect ratio of anchor boxes according to different tracking objects.

#### 3.4.2. Identity Embedding

The function of identity embeddings is to calculate the distance, that is, the similarity between different objects. Learning identity embeddings is learning a function d(.) that makes similar objects close and dissimilar objects far away. For an object $B_{t}^{i}$ marked as i in frame t, the same object $B_{t1}^{i}$ in frame t1, and a different object $B_{t2}^{j}$ marked as j (i≠j) in frame t2, then $d(B_{t}^{i},B_{t1}^{i})<d(B_{t}^{i},B_{t2}^{j})$. Our model produces identity embedding $E\in R^{128\times W_{out}\times H_{out}}$ through the corresponding prediction header. For every object with the center point at (x,y), the embedding is $E_{x,y}\in R^{128}$.

### 3.5. Loss Functions

ViTT’s loss function can be divided into three parts: the loss of classification $L_{C}$, the loss of box offset $L_{R}$, and the loss of identity embedding $L_{E}$. These parts are aggregated using the automatic loss balancing approach [40].

RPN has two tasks: one is to judge whether an anchor box produced based on an anchor point is a foreground object, the corresponding loss function is $L_{C}$; the other is to perform offset regression on candidate boxes, and the corresponding loss function is $L_{R}$.

For $L_{C}$, we use the cross-entropy loss Function (7):

(7) $$L_{C}=\frac{1}{N_{C}}\sum_{i}-\log[p_{i}^{*}p_{i}+(1-p_{i}^{*})(1-p_{i})]$$

where $N_{C}$ is the total number of anchor boxes, $p_{i}$ is the probability that an anchor box is a foreground box, and $p_{i}^{*}$ is the ground truth probability Equation (8):

(8) $$p_{i}^{*}=\left\{\begin{array}{c} \begin{array}{cc} 0 & negative \end{array}\\ \begin{array}{cc} 1 & positive \end{array} \end{array}\right.$$

For $L_{R}$, the loss function is Equation (9):

(9) $$L_{R}=\frac{1}{N_{R}}\sum_{i}p_{i}^{*}R(t_{i}-t_{i}^{*})$$

where $N_{R}$ is the number of all candidate boxes, $t_{i}=\{t_{x},t_{y},t_{w},t_{h}\}$ is the predicted offsets, and $t_{i}^{*}=\{t_{x}^{*},t_{y}^{*},t_{w}^{*},t_{h}^{*}\}$ is the set of ground truth offsets. $t_{i}$ and $t_{i}^{*}$ are shown in Equation (10):

(10) $$\begin{array}{l} t_{x}=(x-x_{a})/w_{a}\\ t_{y}=(y-y_{a})/y_{a}\\ t_{w}=\log(\frac{w}{w_{a}})\\ t_{h}=\log(\frac{h}{h_{a}}) \end{array}\begin{array}{l} t_{x}^{*}=(x^{*}-x_{a})/w_{a}\\ t_{y}^{*}=(y^{*}-y_{a})/y_{a}\\ t_{w}^{*}=\log(\frac{w^{*}}{w_{a}})\\ t_{h}^{*}=\log(\frac{h^{*}}{h_{a}}) \end{array}$$

where x,y,w,h is the center coordinate, width, and height of prediction boxes; $x_{a},y_{a},w_{a},h_{a}$ is the center coordinate, width, and height of anchor boxes; and $x^{*},y^{*},w^{*},h^{*}$ is the center coordinate, width, and height of manually marked objects. R is a smoothing Function (11):

(11) $$R=\left\{\begin{array}{cc} 0.5x^{2} & if\;|x|<1\\ x-0.5 & otherwise \end{array}\right.$$

For $L_{E}$, we choose triple loss [41] $L_{triplet}=\max(f^{T}f^{+}-f^{T}f^{-}+m\arg in,0)$, where $f^{T}$ is an anchor, $f^{+}$ is anchor’s positive sample, and $f^{-}$ is anchor’s negative sample. Ignoring the margin and selecting the most difficult positive example to reduce the sampling space [16], the loss function of identity embedding is defined as Equation (12):

(12) $$L_{E}=-\log\frac{\exp(f^{T}g^{+})}{\exp(f^{T}g^{+})+\sum_{i}\exp(f^{T}g_{i}^{-})}$$

where $g^{+}$ is the class-wise weight of the positive class, and $g_{i}^{-}$ is the negative class’ weight.

For the fusion of multi-task learning loss functions, we adopt the automatic learning strategy [40], and the loss function is defined as Equation (13):

(13) $$L=\sum_{i=1}^{N}\sum_{j=H,B,E}\frac{1}{2}(\frac{1}{e^{s_{j}^{i}}}L_{j}^{i}+s_{j}^{i}).$$

where $s_{j}^{i}$ is a learnable parameter.

## 4. Experiments and Discussion

This section introduces the datasets, evaluation metrics, and implementation details of the ViTT model, analyzes the experimental results, and introduces a series of ablation studies. In addition, this section discusses the limitations of ViTT in tracking small objects. Future research directions also are highlighted in this section, finally.

### 4.1. Datasets and Metrics

#### 4.1.1. Datasets

We evaluated our model on MOT16 [42] benchmarks. MOT Challenge [42] is an authoritative evaluation platform for MOT that provides very accurate annotation data and comprehensive evaluation metrics to evaluate tracking algorithm and pedestrian detector performance. MOT16, whose annotation objects include moving pedestrians and vehicles, has a wealth of images, including different shooting perspectives and camera movements, and contains videos of different weather conditions. There are 14 video sequences in the MOT16 dataset, including 7 training sets with annotation information and 7 test sets. For the model’s training, we use the method of collecting multiple datasets into a large dataset [16]. Among them, PRW [43], CUHK-SYSU [44], MOT17 [42], and CalTech [45] are labeled with object locations and identities. We use these for training the whole model, while ETH [46] and CityPerson [47] are only labeled with object locations, so we use them for training the detection branch.

#### 4.1.2. Evaluation Metrics

MOT Challenge provides the official MOT evaluation metrics, including multiple object tracking accuracy (MOTA), multiple object tracking precision (MOTP), mostly tracked (MT), mostly lost (ML), false negative rate (FN), false positive rate (FP), ID switches (IDs), and ID F1 score (IDF1). The function of MOTP is to quantify the positioning accuracy of detectors, we do not list MOTP as the comparison index of trackers. MOTA is the primary metric to evaluate the comprehensive performance of trackers Equation (14):

(14) $$MOTA=1-\frac{\sum_{t}FP_{t}+FN_{t}+IDs_{t}}{\sum_{t}GT_{t}}$$

where $FN_{t}$ is the false-negative, indicating the number of missed detections, $FP_{t}$ is the false-positive, indicating the number of false detections, $IDs_{t}$ is the number of identity switches, and $GT_{t}$ is the number of ground truth objects. The false-negative rate (FN), false positive rate (FP), and identity switches (IDs) are calculated as follows Equation (15):

(15) $$FN=\frac{\sum_{t}FN_{t}}{\sum_{t}GT_{t}}\;,FP=\frac{\sum_{t}FP_{t}}{\sum_{t}GT_{t}},\;IDs=\frac{\sum_{t}IDs_{t}}{\sum_{t}GT_{t}}$$

The MT is the percentage of successfully tracked objects over most (over 80%) of the time. The ML is the percentage of successfully tracked objects over a small (less than 20%) fraction of the time. The IDF1 measures the difference between the predicted ID and the correct ID:

(16) $$\mathrm{IDF1}=\frac{2\times IDP\times IDR}{IDP+IDR}$$

where IDP is the accuracy of ID, and IDR is the recall rate of ID defined in Equation (17):

(17) $$IDP=\frac{IDTP}{IDTP+IDFN},\;IDR=\frac{IDTP}{IDTP+IDFN}$$

where IDTP is the true-positive of ID, IDFN is the false-negative of ID.

### 4.2. Implementation Details

We used stochastic gradient descent (SGD), which is optimized by Adam [48], with a learning rate of $1\times 10^{-3}$, momentum of 0.9, and a weight decay of $1\times 10^{-4}$. We set the batch size batchsize=32, the random false-positive rate $\lambda_{fp}=0.1$, and the random false-negative rate $\lambda_{fn}=0.2$. Data augmentations included random horizontal flipping, random resizing, and color dithering. We trained all models for 150 epochs, and in the 75th and 112th epochs, the learning rate was decreased by 0.1. We trained and tested our model on the GPU of two RTX 3090. We reshaped the resolution of the input images to 1088 × 608. The encoder consisted of 12 layers with 24 heads, and the patch size was set to 32 × 32. As for track rebirth, we referred to the previous work and suspended unmatched objects temporarily until they were rematched or beyond K = 32 frames. Unless otherwise stated, the following experiments used these default hyperparameters.

### 4.3. Results

We evaluated our baseline model on MOT16 and compared it with other typical trackers: DeepSORT [49], RAR16 [50], TAP [9], CNNMTT [10], and POI [11]. MOT16 Challenge includes public and private detections. Trackers using public detections implement tracking using common detections provided by MOT16 Challenge. The purpose of this protocol is to evaluate the tracker’s similarity measurement and correlation strategy. Trackers using private detections use private detection results to track and have no restrictions on the detection network and training datasets. The purpose of this protocol is to evaluate the overall performance of the trackers, including detection performance. Since our model is not only trained on the training dataset of MOT16, we choose the private detections protocol of MOT16 for evaluation. The models we compared are using private detections protocol, too. Table 1 reports the results of VITT’s evaluation around several key evaluation metrics and its detailed comparison with other trackers. The performance of ViTT is shown in the last row of Table 1: *MOTA* is 65.7%, better than DeepSORT (61.4%), and close to CNNMTT (65.2%). IDF1 is 66.5%, second only to TAP’s 73.5%; MT (39.5%), ML (20.6%), and IDs (706) are also close to the optimal level. ViTT is not optimal for some metrics, but the gap is small, and its performance in the remaining indicators is often excellent. For example, the *MOTA* of ViTT is slightly lower than 66.1% of POI, but in other aspects, ViTT is better than POI. Therefore, we can report that the overall performance of VITT is satisfactory. Speed is an important performance of online trackers and is key to whether a model can handle real-time video. We use frames per second (FPS) to compare the speed of our model. It is worth noting that the other trackers use “Hz” while our tracker uses the detection time plus the association time. Because our model is a multi-task learning model, which parallelizes object detection and embedding learning into a single network, and is much faster than the other separate detection and embedding (SDE) models. As can be seen from Table 1, the speed of ViTT can reach up to 15 frames per second, far exceeding other trackers.

We only provide a simple baseline designed to explore the effectiveness and benefits of our framework without using complex optimizations and tricks. We believe the performance of the model will continue to improve with the addition of some significant optimizations, including replacing the original transformer with improved versions like the Swin Transformer [51]; pre-training the model on large datasets, such as ImageNet-21K [52]; reducing the size of the patches; increasing the input resolution, or applying other standard tweaks. Due to limited hardware and other factors, we did not carry out detailed experiments. Moreover, these optimizations are not the focus of this work.

**Figure 3:**
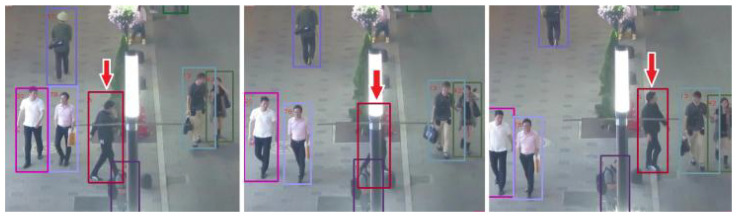
Occlusion tolerance of ViTT. As shown by the arrow, when a person passes through the shelter, ViTT can still track them accurately. The identifiable image in this figure is from the public dataset MOT16.

While testing, we found that ViTT has excellent tolerance for occlusion. Occlusion is one of the main difficulties of MOT, but ViTT based on a transformer can solve this problem. As shown in Figure 3, the model can still track an object accurately when it is almost completely occluded. We know that occlusion can easily cause tracking to fail by causing ID switching. As shown in Table 1, the higher tolerance of occlusion using ViTT is reflected in the lower IDs. We think this is due to the transformer’s powerful ability to combine context, which is difficult for convolution to achieve.

By exploring the relationship between occlusion and IDs, we prove that the lower IDS of this tracker is related to its robustness to occlusion. We selected 3 image sets in the MOT16 test datasets. Each image set consisted of 150 frames of continuous images and contains rich occlusion. We counted the number of occlusions (Nocc) and IDs (NIDs) for each image set. An object is judged occluded when most of its surface area is occluded and then reappears. The reason for blocking out situations that only disappear but do not reappear, such as one going out of frame, is that IDS does not occur in these situations. The number of IDs is obtained from the tracking results. The detailed results are shown in Table 2. As shown in Table 2, the number of IDs of the tracker is positively correlated with the number of occlusions. Under the condition that the number of occlusions is fixed, lower IDS represents higher occlusion robustness. Therefore, ViTT is more effective than CNN-based trackers for complex scenarios including occlusion, such as disorderly people, a jungle with abundant occlusion, etc.

**Figure 4:**
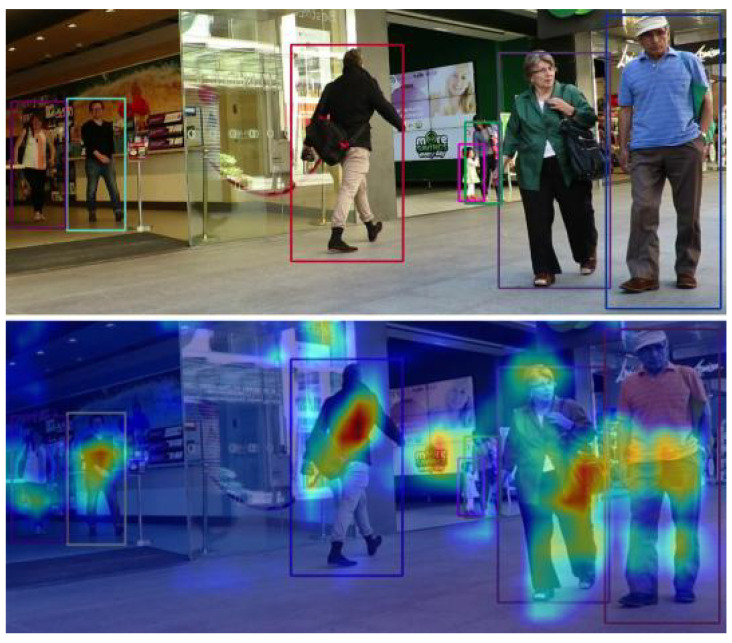
Tracking and its corresponding attention visualization result. We visualized the feature map in front of the tracking head in the form of a hot map. The upper part is a tracking result, and the lower part is the corresponding attention visualization result. The identifiable image in this figure is from the public dataset MOT16.

We visualized the feature map in front of the model’s tracking head in the form of a heat map, which helps to understand the transformer’s advantages more intuitively and confirms our previous analysis. From Figure 4, we can see that the model seems to pay more attention to semantic information, such as walking people, people carrying a bag, and people with their hand(s) in their pants pockets. We believe this is due to the transformer’s powerful ability to connect and understand global context information.

### 4.4. Ablation Study

To explore the working principle of the model, we conducted a series of ablation experiments on ViTT’s core technical contribution: a transformer. Because there are few reference computer vision models based on transformers, we carried out comprehensive ablation experiments on the transformer, including the number of encoder layers and encoder heads, encoder input with different resolutions, multi-layer encoder output aggregation, and training datasets of different sizes.

#### 4.4.1. Number of Encoder Layers and Encoder Heads

**Figure 5:**
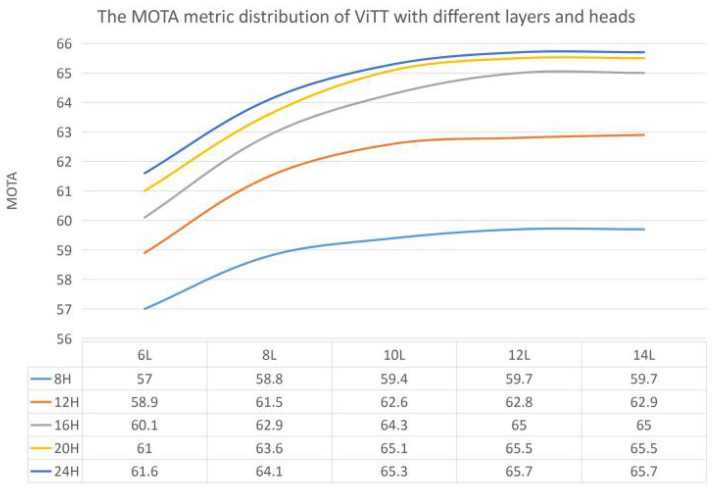
The *MOTA* metric distribution of ViTT with different layers and heads. The “L” and “H” indicate Layers and Heads, respectively.

First, we compared the model’s performance using different numbers of encoder layers and encoder heads. The original transformer encoder had six layers, and each layer had eight heads. Was this size suitable for CV tasks? In order to make the model accurately locate objects, we divided the image into many small patches. Since each object is usually composed of multiple patches, the number of initial layers and heads may not be enough to find the context correlation between them. Intuitively, the more layers and heads the encoder has, the better the model should be. However, due to the complexity of the model and training hardware requirements, it is impossible to increase the number of encoder layers and headers without bounds. We conducted ablation experiments on the number of layers and heads of the encoder and compared the *MOTA* of ViTT with a different number of layers (6, 8, 10, 12, and 14 layers) and different numbers of heads (8, 12, 16, 20, and 24 heads). The experimental results are shown in Figure 5, where the abscissa is the number of layers, and the ordinate is the *MOTA*; different curves represent different numbers of heads.

We find that the number of heads positively correlates but is not strictly proportional to the model’s performance. When the number of heads increases to a particular value, the growth rate of the model’s performance decreases significantly. As shown in Figure 5, as the number of heads increases, the gap between different curves gradually narrows. When the number of layers increases to a specific size, the *MOTA* of the model no longer increases with it. As shown laterally in Figure 5, the curves between the 12 layers and 14 layers are almost horizontal. This is probably because the learning for different patches can be performed in the first few layers of the network, and adding more layers does not contribute substantially to the model’s performance.

#### 4.4.2. Encoder Input with Different Resolutions

For computer vision tasks such as MOT, the precise positioning of objects requires a very detailed image. For our model, the input resolution of the encoder is critical to ensure its performance. However, for patches of fixed size, the computational complexity of the model increases exponentially with increasing resolution. We compared the differences between the models from three different resolutions (576 × 320, 864 × 480, and 1088 × 608). Table 3 lists the experimental results. When the resolution increased from 576 × 320 to 864 × 480, *MOTA* improved by 2.8%; when it is increased to 1088 × 608, *MOTA* continued to improve by 2.3%. The improvement of the encoder’s input resolution is positively correlated with the improvement of model performance. Therefore, model performance can be improved by improving the input resolution (or reducing the patch size) under the condition that the computing force is allowed.

#### 4.4.3. Multi-Layer Encoder Output Aggregation

Inspired by feature pyramid networks (FPN) [53], we wondered if aggregating the encoder output of the middle layers could improve the performance of the model. Therefore, we aggregated the output of layers 4 and 8 with the final output (concatenating them directly and then performing a linear transformation). Table 4 shows a comparison between the aggregated model and the non-aggregated model. As shown in Table 4, the *MOTA* and IDF1 of the aggregated and non-aggregated models are consistent, so the aggregation of middle layers does not improve the model’s performance. This is likely because the model gradually learns the relationship between patches as the number of encoder layers increases, and the output of middle layers has no vital influence on the final judgment.

#### 4.4.4. Training Data Sets of Different Sizes

According to the analysis of Vision Transformer [21], the dataset size has a significant impact on model performance. Its performance on small datasets is inferior to standard classifiers, but as the size of the dataset increases, its performance gradually surpasses other classifiers. ViTT has the same characteristics. We trained the model with different training datasets, and the results are shown in Table 5. When the training set contained “MOT17 [42] + CUHK-SYSU [44] + PRW [43] + ETH [46]” the model’s *MOTA* was only 56.4%. Caltech [45] and CityPersons [47] were added successively so that the *MOTA* value increases by 5.7% and 3.6%, respectively. Consistent with known experience, ViTT still has a significant dependence on the size of the datasets. Only training datasets that are large enough can maximize the performance of ViTT.

### 4.5. Discussion

This section discusses the transformer’s ability to model in a global context, the advantages of our approach in occlusion and complex scenarios, the transformer’s shortcomings in model training and local modeling, and the limitations of our model in tracking small objects. Future research directions also are highlighted in this section.

#### 4.5.1. Limitations of ViTT

VITT is a multi-object tracker based on the transformer. It adopts the image processing method of ViT [21] to divide the image into patches of fixed size. This method has both advantages and disadvantages of the transformer. CNN expands the receptive field and extracts more advanced abstract features through convolution and pooling. The convolution model can not make full use of context information to capture features. Stacking layers of convolution can also make the network too large. The advantage of the transformer is that it uses self-attention to capture global contextual information to establish a remote dependency on the embedding, thus extracting more powerful semantic features [22]. ViTT leverages the strengths of the transformer to show strong robustness in occlusion and complex scenarios. The transformer has the above advantages, but at the same time, there are some disadvantages, more difficult training is an obvious disadvantage of the transformer [21]. The model based on a transformer needs a huge training dataset to achieve excellent performance. ViTT’s training dataset is a large dataset composed of five public datasets. In addition, transformer-based visual models have limitations when dealing with small objects [19]. On the one hand, since the function of the self-attention operator is to model between embedded vectors, the method of dividing the picture into patches cannot understand the semantic information inside the patches well. On the other hand, since the computational complexity of the self-attention operator increases exponentially with the increase in embedded vectors, the size of patches is also limited. Therefore, ViTT has relatively poor performance when tracking small objects.

#### 4.5.2. Future Research Directions

Firstly, considering the limitation of ViTT in tracking small objects, how to effectively model the semantics inside patches or design an efficient transformer to reduce the patch size to improve the performance of the tracker in tracking small objects is a worthy research direction. In addition, this work only explores the advantages of ViTT in complex scenarios involving occlusion. Whether this approach can be expanded to other types of complex scenarios, such as semantic mismatches, category confusion, extreme light conditions, etc., is a worthy research direction, too.

## 5. Conclusions

In this study, we explored the direct application of a transformer for completing complex computer vision tasks. We proposed an MOT model called ViTT based on a transformer. The model replaces a traditional backbone network with a transformer’s encoder, and reinterprets the coding results into spatial feature maps. Compared with traditional convolutional backbones, ViTT can focus on global context information through the encoder’s self-attention layer at the beginning of the network. This multi-task learning model outputs the detection results and appearance embeddings simultaneously in a single shared network, which significantly reduces the calculation requirements of MOT. The competitive performance of the model on MOT16 demonstrated the effectiveness of our method. Our proposed method serves as a reference for new ideas for applying transformers in MOT.

## Figures and Tables

**Table 1:** Comparison with the standard online trackers under the private detections protocol on the MOT-16 benchmark. The arrows indicate low or high optimal metric values.

	*MOTA*↑	IDF1↑	MT↑	ML↓	IDs↓	FPS
DeepSORT	61.4	62.2	32.8%	18.2%	781	<8.1
RAR16	63.0	63.8	39.9%	22.1%	482	<1.5
TAP	64.8	73.5	40.6%	22.0%	794	<8.2
CNNMTT	65.2	62.2	32.4%	21.3%	946	<6.4
POI	66.1	65.1	34.0%	20.8%	805	<6
(ours) ViTT	65.7	66.5	39.5%	20.6%	706	<15

**Table 2:** The relationship between occlusion and IDs. Nocc1–3 represent IDs number of the three image sets, respectively.

	Nocc1 (38)	Nocc2 (74)	Nocc3 (97)
NIDs	7	24	39

**Table 3:** Comparison between different input resolutions of the encoder. The results show that the resolution is essential to the performance of the model.

	*MOTA*	IDF1	MT	ML	IDs
576 × 320	60.6	60.1	32.6%	24.2%	956
864 × 480	63.4	64	36.7%	21.1%	788
1088 × 608	65.7	66.5	39.5%	20.6%	706

**Table 4:** Comparison of aggregated and non-aggregated mid-layer outputs. We aggregate output with layers four layers and layers eight and compare them with the original model. Y is with aggregation, and N is without aggregation.

	*MOTA*	IDF1	MT	ML	IDs
Y	65.7	66.4	39.3%	20.6%	702
N	65.7	66.5	39.5%	20.6%	706

**Table 5:** The comparison of different size training datasets. MCPE is“MOT17 + CUHK-SYSU + PRW + ETH”; MC2PE adds Caltech; and MC3PE adds CityPersons on MC2PE.

	*MOTA*	IDF1	MT	ML	IDs
MCPE	56.4	55.9	28.9%	24.2%	1051
MC^2^PE	62.1	62.0	35.2%	22.1%	843
MC^3^PE	65.7	66.5	39.5%	20.6%	706

## Data Availability

The code of ViTT is available at: https://github.com/jiayannan/VITT.
